# Mini-Margin Nephron Sparing Surgery for Renal Cell Carcinoma 4 cm or Less

**DOI:** 10.1155/2010/145942

**Published:** 2010-08-30

**Authors:** Quanlin Li, Hongwei Guan, Jie Qin, Tao Jiang

**Affiliations:** ^1^Department of Urology, First Affiliated Hospital of Dalian Medical University, 222 zhongshan Road, Dalian 116011, China; ^2^Department of Pathology, First Affiliated Hospital of Dalian Medical University, 222 zhongshan Road, Dalian 116011, China

## Abstract

*Objectives*. To explore the safety and efficacy of mini-margin nephron sparing surgery (NSS) for renal cell carcinoma (RCC) 4 cm or less. *Methods*. Total of 389 cases of RCC 4 cm or less with normal contralateral kidneys were included in the study, including 135 cases treated by mini-margin NSS, 98 by 1 cm-NSS and 156 by radical nephrectomy (RN). The clinical results were followed-up and comparatively analyzed. *Results*. The mean and median margin width for mm-NSS was 2.2 and 2.0 mm (range 0 to 5). Of them, 112 (83.0%) cases had margins of 3 mm or less, and 26 had margins of 0 mm (19.3%). The mean width of margin for 1 cm-NSS was 11.6 mm (median 12, range 10~15). None of the NSS patients had positive surgical margins. The mean follow-up for mm-NSS, 1 cm-NSS and RN patients was 69, 82 and 82 months, respectively. Three mm-NSS patients, two 1 cm- NSS and four RN patients died of non-cancer related causes. Two mm-NSS patient (1.6%) experienced local recurrence. No distant metastasis was detected in all the patients. The over all 5-year survivals for NSS and RN patients were 100%, 100% and 98.7%, respectively (*P* = .950). *Conclusions*. Mini-margin NSS is as safe and effective as 1 cm-NSS and RN in treating early localized RCC 4 cm or less.

## 1. Introduction

Nephron sparing surgery (NSS) has proven to be a safe and effective approach for renal cell carcinoma (RCC), with comparable clinical results to radical nephrectomy, especially for the tumors 4 cm or less, even in patients with completely normal opposite kidneys. For many years, surgical practice for NSS was to have a more than 1 cm margin of normal tissue around the tumor [1, 2]. However, more recent studies show that the width of the margin seems not important. NSS with a smaller margin is as well safe and effective in tumor control [3]. And even pure enucleation is as effective as partial nephrectomy with a rim of healthy parenchyma [4]. 

From January 1998 to December 2008, based on the previous studies in our institute, 135 cases of RCC 4 cm or less in diameter and staged as T1aN0M0 with normal contralateral kidney were treated with 5 mm mini-margin NSS (mm-NSS). The clinical results were followed and compared with 99 cases of NSS with margin 1 cm or more (1 cm-NSS) and 156 cases of radical nephrectomy (RN) for RCC of same stage. The Data were comparatively analyzed to evaluate the safety and efficacy of mini-margin NSS in treating early localized RCC 4 cm or less.

## 2. Materials And Methods

### 2.1. Patient Selection

Only patients with RCC 4 cm or less, with normal contralateral kidney, without lymph node or distant metastasis detected before and during the operation, and clinically Stage T1aN0M0 were included in the analysis. All cases were confirmed by histologic evaluation to be RCC, pathologic Stage T1aN0M0. 

Patients with renal impairment, previous nephrectomy, bilateral or multiple RCCs were excluded. Cases with benign or other type of malignancies such as transitional cell carcinoma rather than RCC revealed at postoperative pathologic examination. And those with nodal or distant metastasis and fat invasion detected before the operation by imaging technique or during the operation by frozen section analysis were also excluded. However, patients with nodal or distant metastasis detected after partial nephrectomy were not excluded. 

From January 1998 to December 2008, 395 cases of RCC 4 cm or less undergone surgical treatment in author's institute, including 140 cases treaded with mm-NSS, 98 with 1 cm-NSS and 157 with RN. 

Patients underwent 1 cm-NSS and RN were mainly from the earlier time, as in recent years most cases with RCC T1a were preferably treated with mm-NSS. Central location alone or invasion to the calyx was not a sufficient reason to perform RN. 

Thus, there were 5 patients (mm-NSS) with synchronous bilateral RCC, and 1 patient (RN) with positive regional node revealed by pathologic analysis were excluded from the study.

### 2.2. Histopathology

All cases were staged, classified, and graded according to TNM criteria 2002, UICC new classification system 1997 and Fuhrman nuclear grading system. Only patients with histologically confirmed RCC were included in the analysis.

### 2.3. mm-NSS

In the remaining 135 cases of NSS, there were 106 males and 29 females, with an average age of 57 (median 56, range 41~73). All the cases were with normal contralateral kidneys. Of the 135 tumors, 125 tumors were peripherally located (with 105 cases at upper or lower pole of the kidneys and 20 at the middle part) whereas 10 tumors were centrally located. 

Preoperatively, all the patients treated with mini-margin NSS were informed of the surgery and asked to sign the permission paper. The surgical procedure for mini-margin NSS was as described in previous reports [5]. The kidney capsule was sharply incised less than 5 mm away from tumor pseudocapsule and the tumor was enucleated with an anticipated margin of less than 5 mm. Renal hypothermia with ice slush was used when a prolonged ischemia time (longer than 30 minutes) was anticipated, such as for centrally located tumors. To prevent renal ischemic damage, all patients were vigorously hydrated and infused with mannital a few minutes before vessel occlusion and furosemide was adopted following clamp removing. 

After removing the tumor, the sample was meticulously checked for gross margin status. When no renal parenchyma was present outside the pseudocapsule, additional thin layer of renal parenchyma was resected for margin pathological evaluation. The frozen section analysis included the pathologic diagnosis, margin width, and determination of possible residual tumor. The maximal and minimal distances from the cut edge of the renal parenchyma to the tumor pseudocapsule were measured for each case. When no renal parenchyma was present outside the pseudocapsule, the margin width was recorded as zero.

### 2.4. 1 cm-NSS

Ninety-eight cases of RCC 4 cm or less were treated with 1 cm-NSS, including 75 cases of male and 23 of female, with an average age of 60 (median 59, range 46~70). All the patients were with unilateral RCC and normal contralateral kidneys. Surgical procedure of 1 cm-NSS was similar to mm-NSS, but tumor was incised 1 cm or more away from pseudocapsule. Margin evaluation was done as described for mm-NSS.

### 2.5. RN

In the 156 cases of RN included, there were 117 males and 39 females, with an average age of 59 (median 59, range 45~75). All the patients were with unilateral RCC and normal contralateral kidneys. Surgical procedure for RN was routinely performed as standard technique.

### 2.6. Followup

The first time followup was executed 3 months after NSS or RN, and CT scanning was done for evaluation of the kidney's morphologic changes and for thereafter reference in NSS patients but not in RN. The regular followup afterward for both groups of patients was conducted every 3~6 months in refer to renal function, urine routine test, kidney ultrasound, and chest X-ray. CT scan or MRI of the kidneys and chest was performed annually, or at any time in cases of clinical suspicion by ultrasonography or X-ray.

### 2.7. Statistical Analyses

Comparisons of features between patients treated with NSS and RN were evaluated using the chi-square, Fisher's exact and *t-*test. Overall and cancer-specific survivals were estimated using Kaplan-Meier method. Statistical analyses were performed using SPSS 13.0 software package. All tests were 2-sided and *P* < .05 was considered statistically significant.

## 3. Results

The clinicopathologic characteristics of the three groups of patients are reported in Table 1. The groups were well matched for patients' sex, tumor size, nuclear grade, histologic classification. The mean age of RN patients was higher than that of NSS patients (*P* = .018) as surgeons were more likely to choose younger patients for conservative surgery. 

All surgical procedures were technically successful either for NSS or RN. The mean duration of the whole surgical procedure and mean estimated blood loss were 85 minutes (range 60~90) and 60 mL (range 50~80) for mm-NSS, 95 minutes (range 70–110) and 70 mL (50–100) for 1-cm NSS, and 60 minutes (range 50~80) and 20 mL (range 10~50) for RN. No intraoperative complications occurred for both the group of patients. None of the patients died within the first 30 days after surgery. 

Staging lymphadenectomy was performed in 7 cases when enlarged regional lymph nodes were found during surgery. Malignancy was detected in one RN case (nodal metastasis) on pathologic analysis and this case was excluded from the study. 

### 3.1. mm-NSS

Renal vessel occlusion was used in 108 patients and simple parenchyma compression in 27 for bleeding control. Renal hypothermia with ice slush was used in 32 patients (23.7%), but it was unnecessary in most cases. The mean hypothermia ischemia time was 21 minutes (median 22, range 20 to 35). Warm ischemia surgery was used in 103 patients (76.3%). The mean warm ischemia time was 16 minutes (median 18, range 7 to 22).

The mean and median diameters of the tumors were 3.3 and 3.5 cm (range 1.0 to 4.0). All cases were stage T1aN0M0. Using the Fuhrman nuclear grading system, 53 (39.3%) of the tumors were G1, 77 (57.0%) G2, 5 (4.3%) G3, and none G4. Renal clear cell carcinoma was confirmed in 105 patients (77.8%), papillary RCC in 18 (13.3%), chromophobe renal cell carcinoma in 9 (6.7%), and multicystic clear cell renal cell carcinoma in 3 (2.2%). 

None of the patients had positive surgical margins detected on pathologic examination, frozen section analysis, or final paraffin section analysis. The minimal margin was always at the bottom of the tumor, where the mean actual margin thickness was 2.2 mm (median 2.0, range 0 to 5). The maximal margin was always at the area of the renal capsule, where the mean actual margin was 4.5 mm (median 5.0, range 4 to 6). If considering the minimal margin only in the analysis, all the cases had a margin of 5 mm or less, 112 (83.0%) had margins of 3 mm or less, and 26 had margins of 0 mm (19.3%). 

One patient (0.7%) experienced slight secondary gross hematuria 2 weeks after surgery but no arteriovenous fistula or pseudoaneurysm was detected on computed tomography angiography. This patient was treated conservatively without the need for blood transfusion or surgical intervention. No major complications such as urinary leakage, urinoma, or hemorrhage requiring reoperation occurred.

### 3.2. 1 cm-NSS

The mean and median diameter of the 98 tumors treated by 1 cm-NSS was 2.9 and 3.2 cm (range 1.0~4.0). All cases were stage T1aN0M0. The Fuhrman nuclear grading was G1 in 31 cases (31.6%), G2 in 65 (66.3%), G3 in 3 (3.1%), and none G4. Renal clear cell carcinoma was confirmed in 78 (79.6%) cases, papillary RCC in 15 (15.3%), chromophobe RCC in 4 (4.1%), and multicystic clear cell renal cell carcinoma in 1 (1.0%).

None of the patients had positive surgical margins detected pathologically. The mean margin thickness was 11.6 mm (median 12.0, range 10 to 15). 

Collecting system open occurred in 25 patients and urinary leakage developed in 3. Two of them were cured conservatively and 1 was cured by Double-J stent installation. No serious hemorrhage requiring reoperation occurred. NSS had to be turned to radical nephrectomy in 2 patients with centrally located tumors due to damage of main blood supply vessel or renal pelvis which was unable to repair. The overall complication rate was (5/98, 5.1%). The average size of these tumors was 3.6 cm (range 3.0~4.0).

### 3.3. RN

In the 156 cases of RCC treated by RN, the mean and median diameters of the tumors were 3.0 and 3.3 cm (range, 1.5~4.0). All cases were stage T1aN0M0. On the basis of Fuhrman nuclear grading system, 56 (35.9%) of the tumors were G1, 95 (60.9%) G2, 5 (3.4%) G3, and none G4. Renal clear cell carcinoma was confirmed in 125 (80.1%) cases, papillary RCC in 23 (14.7%), and chromophobe RCC in 8 (5.1%). No major complication occurred.

### 3.4. Followup and Survivals

Until last evaluation in December 2009, the mean followup for mm-NSS, 1 cm-NSS, and RN groups was 69 months (median 73, range 15 to 130), 82 months (median 82, 60~103), and 82 months (median 78, range 23 to 134), respectively. Three mm-NSS patients died 77, 88 and 104 months after operation, two 1 cm-NSS patients died 81, 95 months after surgery and four RN patients died 47, 66, 76, and 106 months post operation, but none of them cancer related. Two mm-NSS patient (1.5%) experienced local recurrence, including one of ectopic recurrence in the same kidney and one of tumor bed recurrence. The ectopic recurrence occurred 32 months after NSS at a different site away from the original tumor bed. The original tumor of 3.5 cm (clear cell type and G2) was located at the lower pole of the kidney, and the recurrent tumor (also clear cell type and G2) was detected at the upper pole 32 months after NSS. The case of tumor bed recurrence was detected 25 months after mm-NSS and the margin width of this case was 4 mm. Both patients were cured by remedial radical nephrectomy in respect of the patient's choice, with no evidence of disease at the last followup visit. No local recurrence occurred in patients treated by 1 cm-NSS and RN. No distant metastasis was detected in the three groups of patients.

**Figure 1:**
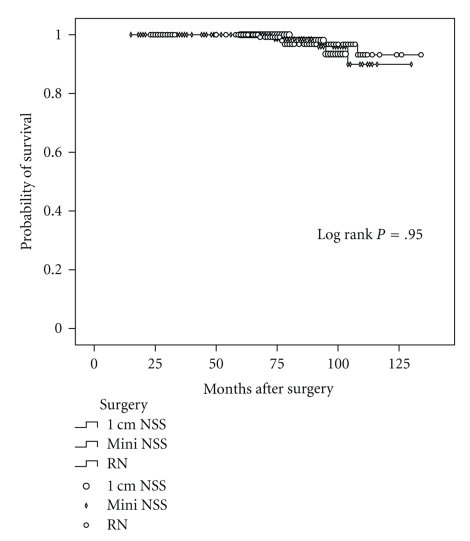
Overall survival for 135 patients treated with mm-NSS, 98 patients with 1 cm-NSS and 156 patients with RN.

Using Kaplan-Meier survival analyses, the overall 5-year survivals for mm-NSS, 1 cm-NSS, and RN patients were 100%, 100% and 98.7% (see Figure 1), Log Rank statistic 0.102, *P* = .950. Cancer specific survivals were not estimated as none of patients' death in both groups was cancer-related. Recurrence-free survival analysis was also not performed as only two mm-NSS patients had local recurrence during followup.

## 4. Discussion

In recent decades, the excellent cancer specific survival which has been reported for imperative NSS has led to the increased use of NSS in cases without imperative indications for conservative surgery. There are extensive reports to support elective NSS for renal masses 4 cm or smaller in greatest dimension [6–8]. Cancer-specific and metastases-free survival are comparative between patients treated with NSS and RN for small early RCC [9, 10], and complication rates, morbidity and mortality are similar for NSS and RN [11, 12], while NSS provides better preservation of renal function than RN [9]. 

What must be minimized with nephron sparing surgery is the possibility of local recurrences. It has been suggested that at least some local recurrence after partial nephrectomy may be due to residual tumor cells on the tumor bed. Based on the assumption, resection of the tumor with a 1-cm margin of normal-appearing parenchyma around the tumor had been considered the standard surgical technique for NSS for many years [1, 2]. However, the size of the surgical margin that should be removed with the tumor remains controversial. An optimal margin will guarantee complete tumor removal as well as keep local recurrence rates to a minimum. An over-resected margin could increase the surgical difficulty and compromise residual renal function, especially in the case of a solitary kidney. It could also increase the morbidity of any surgical complications. In a retrospective study of 69 patients who had undergone NSS, Castilla et al. [13] found, after a mean followup of 8.5 years, that a histologic tumor-free resection margin was sufficient to achieve complete local RCC control and that the width of the resection margin did not correlate with long-term disease progression. Piper et al. [14] reported that a 1-mm margin of normal tissue is necessary to prevent recurrence in a study of 67 patients with a mean followup of 60 months. Accordingly, Sutherland et al. [15] investigated the effects of surgical margin size on recurrence in 44 patients who had undergone partial nephrectomy. The mean and median negative margin was 2.5 mm and 2 mm (range 0.5 to 7), respectively. They concluded that as long as the tumor bed was free of residual tumor, the margin size was irrelevant and did not correlate with disease progression during NSS for low-stage RCC. Recently, Akçetin et al. [3] also found that an increased margin was not clinically related to patients' survival if the margin was greater than 2 mm. Similar results were reported by Timsit et al. [16] and Berdjis et al. [17]. In a prospective study of Stage pT1a RCC treated with radical nephrectomy, Li et al. [18] found positive cancer lesions beyond the pseudocapsule in 19.5% of patients, with an average distance from the primary tumor of 0.5 mm (standard deviation 1.3, range 0 to 5.0), and concluded that when partial nephrectomy was performed, a 5-mm margin could be enough to prevent local recurrence. More recent studies even showed that pure enucleation is as effective as partial nephrectomy with a rim of healthy parenchyma [4]. 

In the present study, mm-NSS was performed using a margin of less than 5 mm, with most patients having a margin of less than 3 mm (83.0%), including 26 patients with 0-mm margins at the tumor bottom (19.3%). The median margin was only 2 mm, but no positive surgical margin was detected. Also, the long-term followup showed a comparative overall survival with 1 cm-NSS and radical nephrectomy. And no patient in three groups died of cancer-related causes and no metastasis detected during followup. Only 1 NSS patient developed local recurrence at tumor bed. One case had tumor recurrence in a different site away from the original tumor bed. This case of ectopic recurrence might have been due to tumor multifocality. Our study also shows a significant lower complication rate in mm-margin NSS than that in 1 cm-NSS. 

These data show that mini-margin NSS with a less than 5-mm margin can effectively achieve local tumor resection with excellent long-term patient survival for those with RCC of 4 cm or less (T1aN0M0), while not increasing the local recurrence rate. Additionally, it could imply that the size of the surgical margin is not relevant and not related to disease progression, as long as the tumor is completely excised. Moreover, further potential advantages of mini-margin NSS are in favor of preservation of renal parenchyma, and with a lower incidence of major blood supply vessel and collecting system damage, the latter evolving toward urinary leakage, urinoma, and urinary fistula if undetected and not repaired during surgery, especially when tumor locates centurally.

## 5. Conclusions

Mini-margin NSS is as safe and effective as 1 cm-NSS and RN in treating early localized RCC 4 cm or less. It provides excellent renal function preservation, favorable long-term progression-free survival, lower complication rate, and is not associated with an increased risk of local recurrence.

## Figures and Tables

**Table 1:** Comparisons of the clinical and pathological features.

	mm-NSS	1 cm-NSS	RN	*P* Value
No. of Pts	135	98	156	

Pts age at surgery (yr)				
Mean	57	60	59	**.018**
Median (range)	56 (41~73)	59 (46~70)	59 (45~75)	
No. male	106 (78.5%)	75 (76.5%)	117 (75.0%)	.779
Tumor size (cm)				
Mean	3.3	2.9	3.0	.513
Median (range)	3.5 (1.0~4.0)	3.2 (1.0~4.0)	3.3 (1.5~4.0)	
Fuhrman nuclear grade				
G1+2	130 (96.3%)	96 (98.0%)	151 (96.8%)	.764
Histologic classification				
Clear cell RCC	105 (77.8%)	78 (79.6%)	125 (80.1%)	.880
Surgical margin (mm)				
Mean (median, range)	2.2 (2.0, 0~5)	11.6 (12, 10~15)	—	.000*
Complication rate	0.7% (1/135)	5.1% (5/98)	—	.049*
Followup (months)				
Mean (median, range)	69 (73, 15~130)	82 (82, 60~103)	82 (78, 23~134)	**.000**

RCC = renal cell carcinoma, pts = patients; *compared between mm-NSS and 1 cm-NSS groups.

## Abbreviations and Acronyms

RCC: Renal cell carcinoma
NSS: Nephron sparing surgery
RN: Radical nephrectomy.
